# Gal-geun-dang-gwi-tang improves diabetic vascular complication in apolipoprotein E KO mice fed a western diet

**DOI:** 10.1186/1472-6882-14-453

**Published:** 2014-11-22

**Authors:** So Min Lee, Yun Jung Lee, Jung Hoon Choi, Min Chul Kho, Jung Joo Yoon, Sun Ho Shin, Dae Gill Kang, Ho Sub Lee

**Affiliations:** Professional Graduate School of Oriental Medicine and College of Oriental Medicine, Wonkwang University, Shinyong-dong, Iksan, Jeonbuk, 570-749 Republic of Korea; Hanbang Body-fluid Research Center, Wonkwang University, Iksan, Republic of Korea; Department of Internal Medicine, College of Oriental Medicine, Wonkwang University, Iksan, Republic of Korea; Brain Korea (BK) 21 plus team, Professional Graduate School of Oriental Medicine, Wonkwang University, Iksan Jeonbuk, 540-749 Republic of Korea; Hamsoa Oriental Medical Clinic, Yeonhyang-dong, Suncheon Jeonnam, 540-952 Republic of Korea

**Keywords:** Gal-geun-dang-gwi-tang, Diabetes, Atherosclerosis, Insulin resistance, eNOS

## Abstract

**Background:**

Gal-geun-dang-gwi-tang (GGDGT), an herbal medicine, is used to treat hypertension, stroke, and other inflammatory disorders in the clinical setting. Recently, GGDGT was recognized by the Korea Institute of Oriental Medicine. This study aimed to evaluate the effects of GGDGT in a diabetic atherosclerosis model using apolipoprotein E knockout (ApoE-/-) mice fed a Western diet.

**Methods:**

The mice were divided into four groups: control group, C57BL6J mice receiving a regular diet (RD); ApoE-/- group, ApoE-/- mice receiving a Western diet (WD); rosiglitazone group, ApoE-/- mice receiving rosiglitazone (WD + 10 mg · kg^-1^ · day^-1^); GGDGT group, ApoE-/- mice receiving GGDGT (WD + 200 mg · kg^-1^ · day^-1^).

**Results:**

Treatment with GGDGT significantly improved glucose tolerance and plasma lipid levels. In addition, GGDGT ameliorated acetylcholine-induced vascular relaxation of the aortic rings. Immunohistochemical staining showed that GGDGT suppressed intercellular adhesion molecule (ICAM)-1 expression; however, expression of endothelial nitric oxide synthase (eNOS) and insulin receptor substrate (IRS)-1 were restored in the thoracic aorta and skeletal muscle, respectively.

**Conclusions:**

These findings suggest that GGDGT attenuates endothelial dysfunction via improvement of the nitric oxide (NO)-cyclic guanosine monophosphate (cGMP) signalling pathway and improves insulin sensitivity in diabetic atherosclerosis.

**Electronic supplementary material:**

The online version of this article (doi:10.1186/1472-6882-14-453) contains supplementary material, which is available to authorized users.

## Background

Migration of circulating monocytes into the vessel wall is an important step in the development of diabetic atherosclerosis, a chronic inflammatory condition. Hypercholesterolemia produces numerous functional and structural alterations in the vascular walls and leads to the development of atherosclerosis [1]. Insulin resistance, an important feature of metabolic diseases, serves as a common pathophysiological basis shared by cardiovascular diseases such as diabetes, hyperlipidemia, hypertension, and hyperglycemia [2, 3]. Production of local reactive oxygen species can increase low-density lipoprotein (LDL) oxidation, local inflammation, vascular adventitial fibroblast proliferation, and extracellular matrix synthesis. Local reactive oxygen species can also directly activate nuclear factor kappa B (NF-κB) and stimulate the expression of NF-κB-dependent genes, including the genes of pro-inflammatory factors related to atherosclerosis, such as intercellular adhesion molecule (ICAM)-1. Increased expression of pro-inflammatory genes advances the atherosclerotic process and vascular remodelling [4, 5].

A high-cholesterol Western diet causes atherosclerotic plaque formation, vascular inflammation, and lipid metabolism disorders and leads to hyperlipidemia and insulin resistance, which are characterized by high levels of serum triglycerides and total cholesterol [6–8]. Higher serum LDL results in increased risk of atherosclerosis and cardiovascular diseases, whereas high-density lipoprotein (HDL) protects against atherosclerosis [9]. Many epidemiological, clinical, and experimental studies have indicated that reducing high serum LDL is an effective way to prevent atherosclerosis and cardiovascular diseases [10]. Moreover, vascular tone is an important factor in the regulation of arterial blood pressure. Changes in vascular smooth muscle tone and the internal diameter of vessels can profoundly alter tissue perfusion and impair the ability of arteries to respond to vasodilators and vasoconstrictors. The endothelium-dependent vasorelaxation that is induced by acetylcholine (ACh) is mediated by nitric oxide (NO), which acts through soluble guanylyl cyclase and cyclic guanosine monophosphate (GMP). Thus, this phenotypic change appears to result from a decline in NO bioavailability due to impaired NO biosynthesis and inactivation of NO by superoxide, which leads to hypertension. A recent study showed that ACh-induced relaxation was decreased in apolipoprotein E knockout (ApoE-/-) mouse aortas, but the dysfunction was strictly correlated with the development and size of atherosclerotic plaques. This indicates that the endothelial defect is not determined by hypercholesterolemia alone, but is predominantly associated with plaque formation [11, 12]. These impaired vascular responses have been found in hypercholesterolemic animals [13, 14] and humans [15]. Impaired relaxation of the aorta in response to ACh in obese rats is a consequence of endothelial dysfunction [16].

In traditional Oriental medicine, various herbs or herbal prescriptions have long been used to treat vascular disorders such as stroke or atherosclerosis [17]. Gal-geun-dang-gwi-tang (GGDGT), a Korean herbal medicine, has traditionally been prescribed for the treatment of diabetes. GGDGT has been in clinical use for many years, and the Korea Institute of Oriental Medicine included GGDGT in a compilation published in 2004 [18]. However, the mechanism of GGDGT has yet to be clarified. Therefore, we used ApoE-/- mice as an animal model of diabetic atherosclerosis [19–21] and rosiglitazone as a positive control to investigate the beneficial effect of GGDGT on vascular dysfunction and metabolic disorders.

## Methods

### Preparation of GGDGT

The 17 herbal ingredients of GGDGT were purchased from the Herbal Medicine Cooperative Association (Iksan, Korea). A voucher specimen (No. HBA091) has been deposited in the Herbarium of the Professional Graduate School of Oriental Medicine, Wonkwang University (Korea). The ingredients of GGDGT include *Pueraria lobata* Ohwi, *Glycyrrhiza uralensis* Fischer, *Angelica gigas* Nakai, *Liriope platyphylla* Wang et Tang, *Paeonia japonica* (Makino) Miyabe & Takeda, *Cornus officinalis*, *Chaenomeles sinensis* Koehne, *Rehmannia glutinosa* Liboschitz var. purpurea Makino, *Nelumbo nucifera* Gaertner, *Prunus mume* Sieb et Zucc, *Schisandra chinensis* (Turcz.) Baill, *Phyllostachys nigra* Munro var. henosis Stapf, *Anemarrhena asphodeloides* Bunge, *Cnidium officinale* Makino, *Asparagus cochinchinensis* (Lour.) Merr, *Trichosanthes kirilowii* Maximowicz, and *Cyperus rotundus* L. The herbal mixture was boiled and then dried to form granules.

### Experimental animals

The animal procedures were in strict accordance with the 1985 (revised 1996) guidelines for the care and use of laboratory animals of the U.S. National Institutes of Health and were approved by the Institutional Animal Care and Utilization Committee for Medical Science of Wonkwang University. Six-week-old male ApoE-/- C57BL6J mice (n = 36) and normal C57BL6J mice (n = 12) were obtained from Central Laboratory Animal (Seoul, Korea). Mice were started on a Western diet (WD) containing 0.15% cholesterol and providing 42% calories from fat (TD 88137; Harlan Teklad, Madison, WI) at 6 weeks of age, and maintained on this diet for an additional 12 weeks. Throughout the experiments, all animals had unrestricted access to water. After two weeks of acclimatization, animals were randomly divided into four groups (n = 12 per group). In the control group, C57BL6J mice received a regular diet (RD). The peroxisome proliferator-activated receptor-γ (PPAR-γ) agonist, rosiglitazone, was chosen as a positive control. Rosiglitazone is an anti-diabetic drug for the treatment of type 2 diabetes [22]. In the rosiglitazone group, ApoE-/- mice received rosiglitazone (WD + 10 mg · kg^-1^ · day^-1^). In the GGDGT group, ApoE-/- mice received GGDGT (WD + 200 mg · kg^-1^ · day^-1^). At the end of the study, the mice were fasted overnight and their organs were collected.

### Measurement of blood pressure

Systolic blood pressure (SBP) was determined by a tail-cuff plethysmography method and recorded with an automatic sphygmotonograph (Muromachi Kikai, Tokyo, Japan). At least eight determinations were made in every session, and the mean of the lowest five values within 5 mm Hg was taken as the systolic blood pressure level.

### Oral glucose tolerance test

At the end of week 2 of the experimental period, the mice were fasted overnight before administration of glucose (1.5 g/kg). Blood samples were collected from the tail at various time points (0–120 min) after glucose loading, and blood glucose levels were measured with a Onetouch Ultra blood glucose meter and test strip (LifeScan, Inc., Milpitas, CA).

### Analysis of plasma biochemicals

The blood glucose concentration was measured at 1, 4, 8, and 11 weeks of GGDGT supplementation. The blood glucose concentration was measured with whole blood obtained from the tail veins after withholding food for 6 h using a Onetouch Ultra blood glucose meter and test strip (LifeScan, Inc.). Plasma levels of total cholesterol (T-Cho) and triglycerides (TG) were enzymatically measured using commercially available kits (Arkray Factory, Inc., Kyoto, Japan). Plasma insulin levels were determined by an ultrasensitive mouse insulin ELISA (enzyme-linked immunosorbent assay) (ALPCO Diagnostics, Salem, NH), and plasma leptin levels were measured using a mouse leptin ELISA (Crystal Chem, Downers Grove, IL).

### Recording of isometric vascular tone

Vascular tone was determined as previously described by Kang et al. [23]. At the end of the experiment, the mice were sacrificed by decapitation. The thoracic aorta was rapidly and carefully dissected and placed in ice-cold Krebs solution (118 mM NaCl, 4.7 mM KCl, 1.1 mM MgSO_4_, 1.2 mM KH_2_PO_4_, 1.5 mM CaCl_2_, 25 mM NaHCO_3_, and 10 mM glucose, pH 7.4). The aortas were removed free of connective tissue and fat and cut into rings with a width of approximately 3 mm. All dissecting procedures were done with extreme care to protect the endothelium from inadvertent damage. The aortic rings were suspended by means of two L-shaped stainless steel wires inserted into the lumen in a tissue bath containing Krebs solution at 37°C. A gas mixture of 95% O_2_ and 5% CO_2_ was continuously bubbled through the bath. The baseline load placed on the aortic rings was 1.0 g. Changes in isometric tension were recorded using a Grass model FT 03 force displacement transducer (Grass Technologies, Quincy, MA) connected to a model 7E polygraph recording system (Grass Technologies). The aortic rings were washed every 10 min with Krebs solution until the tension returned to the basal level. After being stabilized, the aortic rings were contracted with phenylephrine (1 μM), a selective α1-adrenergic receptor agonist, to obtain a maximal response, and then a concentration-dependent response curve to ACh, a vasodilator that stimulates endothelial release of NO, or sodium nitroprusside (SNP), an exogenous NO donor, was determined in the thoracic aorta.

### Histological examination

Aortas isolated from all groups were fixed with 10% (v/v) formalin and embedded in paraffin (50 mM potassium paraffin). Then cross-sections (6 μm) of the aortic arch in each group were stained with haematoxylin and eosin (H&E) [24]. The Oil-red O stain was used to identify lipid accumulation in the plaque. Assessments were made using *en face* preparations of whole descending aortas as previously described [25]. Briefly, blood was removed from dissected descending thoracic aortas by perfusion with phosphate-buffered saline (PBS). Then the aortas were opened longitudinally with extremely fine micro-scissors (George Tiemann & Co, Hauppauge, NY) and turned inside-out to expose the endothelium. The dissected aortas were washed in distilled water and then in 100% propylene glycol and stained with 1% Oil-red O (Sigma Chemical Co., St. Louis, MO) for 25 min at room temperature. Aortas were washed again in 60% propylene glycol and distilled water, mounted on glass slides, and coverslipped using an aqueous medium (Aquatex; Merck, Darmstadt, Germany). Forty fields in three individual sections were randomly selected from the H&E and Oil-red O stained areas. Representative sections were photographed, and quantification of immunohistological staining was conducted by Axiovision 4 imaging/archiving software (Carl Zeiss, Jena, Germany).

### Immunohistochemistry

Sections were stained before they were incubated with 5% normal goat serum for 10 min at room temperature to reduce nonspecific background staining. ICAM-1 (Zymed Laboratories Inc., San Francisco, CA) and endothelial nitric oxide synthase (eNOS) antibody (Oncogene, Cambridge, MA) were applied as a 1:500 dilution and incubated in humidified chambers overnight at 4°C. All slides were then sequentially incubated with biotinylated secondary antibody and horseradish peroxidase–conjugated streptavidin, both for 10 min at room temperature. Peroxidase activity was visualized by the 3-amino-9-ethylcarbazole (AEC) substrate-chromogen system (Zymed), which resulted in brownish-red staining, as previously described by Matsuda et al. [26]. Representative sections were photographed, and quantification of immunohistological staining was conducted by Axiovision 4 imaging/archiving software (Carl Zeiss).

### Western blot analysis

Samples of thoracic aorta were homogenized in a buffer consisting of 250 mM sucrose, 1 mM EDTA, 0.1 mM phenylmethylsulfonyl fluoride (PMSF), and 20 mM potassium phosphate buffer, at pH 7.6. Tissue homogenates (40 μg of protein) were separated by 10% sodium dodecyl sulfate (SDS)-polyacrylamide gel electrophoresis and transferred to nitrocellulose paper. Blots were then washed with H_2_O, blocked with 5% skimmed milk powder in TBST (10 mM Tris–HCl [pH 7.6], 150 mM NaCl, 0.05% Tween-20) for 1 h, and incubated with the appropriate primary antibody at dilutions recommended by the supplier. Then the membrane was washed, and primary antibodies were detected with goat anti-rabbit-IgG conjugated to horseradish peroxidase, and the bands were visualized with enhanced chemiluminescence (Amersham, Buckinghamshire, UK). Protein expression levels were determined by analysing the signals captured on the nitrocellulose membranes using the Chemi-doc image analyser (Bio-Rad, Hercules, CA).

### Statistical analysis

All the experiments were repeated at least three times. The results were expressed as mean ± standard error of the mean (SEM). All statistical analysis was performed using one-way analysis of variance (ANOVA) followed by the Student *t*-test using Sigma Plot (version 10.0). Values of p < 0.05 were considered statistically significant.

## Results and discussion

The present study provides the first evidence that GGDGT significantly attenuates diabetic atherosclerotic development through the inhibition of endothelial dysfunction and insulin resistance. GGDGT, based on its effective combination of medicinal herbs, is included among contemporary prescriptions and has been widely used for many years in the clinical setting. The Korea Institute of Oriental Medicine has published a compilation of herbal prescriptions (‘*Usugyeongheombangjip*, an excellent collection of prescriptions used in the clinical experience in Korea’) that have shown excellent effects in the treatment of diabetic atherosclerosis in modern diseases.

### Effects of GGDGT on metabolism and blood pressure

Hypertension is not always uniformly observed in obesity induced by elevated-fat diets in animal models of endothelial dysfunction [27, 28]. However, we observed an elevation in SBP in WD-fed ApoE-/- mice. We measured the mean SBP using a tail-cuff technique. As shown in Table 1, WD-fed ApoE-/- mice showed significantly increased body weight compared with RD-fed control mice, while body weight was significantly decreased by treatment with GGDGT. Moreover, prior to the start of the experiment (week 0), the mice were not fed a Western diet. Initiation of feeding with a WD resulted in increased SBP in the ApoE-/- mice. However, in the ApoE-/- mice treated with GGDGT, the SBP did not change between week 0 and week 11. Therefore, we think that GGDGT suppressed the increase in blood pressure observed in WD-fed ApoE-/- mice.

**Table 1:** **Effects of GGDGT on body weight, water intake, food intake, blood glucose, and blood pressure levels in ApoE-/- mice**

	Weeks				
		0	4	8	11
Body weight (g)	Control	22.33 ± 0.67	24.08 ± 0.79	25.73 ± 0.79	27.42 ± 0.87
	ApoE -/-	22.57 ± 0.65	28.23 ± 0.70**	33.60 ± 1.01**	37.55 ± 0.90**
	Rosiglitazone	22.07 ± 0.59	27.25 ± 0.97#	31.77 ± 1.22##	34.47 ± 1.35##
	GGDGT	21.45 ± 0.35	25.42 ± 0.56#	29.95 ± 0.95##	32.43 ± 1.16##
Water intake (g/day)	Control	37.63 ± 2.19	44.68 ± 2.15	39.38 ± 3.00	50.59 ± 0.67
	ApoE -/-	38.98 ± 2.92	31.31 ± 2.68	23.21 ± 1.97	30.53 ± 3.07
	Rosiglitazone	38.74 ± 3.01	30.08 ± 0.72	25.05 ± 0.71	22.68 ± 1.28
	GGDGT	29.62 ± 2.11	23.18 ± 1.82	21.30 ± 1.53	24.45 ± 2.00
Food intake (g/day)	Control	19.87 ± 0.45	24.33 ± 0.93	20.85 ± 0.52	20.65 ± 0.55
	ApoE -/-	18.06 ± 0.31	18.37 ± 0.26	17.58 ± 0.49	15.98 ± 0.42
	Rosiglitazone	16.58 ± 0.35	15.00 ± 0.22	14.02 ± 0.62	12.77 ± 0.79
	GGDGT	17.60 ± 0.52	16.24 ± 0.33	16.17 ± 0.20	14.11 ± 0.43
Glucose (mg/dl)	Control	119.60 ± 1.63	114.00 ± 5.30	102.00 ± 2.48	113.67 ± 3.48
	ApoE -/-	123.80 ± 9.28	104.00 ± 6.66	114.60 ± 6.87	138.25 ± 3.42**
	Rosiglitazone	127.33 ± 7.54	95.25 ± 14.44	097.75 ± 2.75	113.75 ± 3.25##
	GGDGT	129.33 ± 4.37	98.67 ± 1.48	107.50 ± 3.27	118.20 ± 3.64##
Systolic blood pressure (mmHg)	Control	102.06 ± 1.47	103.16 ± 1.2	104.16 ± 1.33	109.00 ± 0.91
	ApoE -/-	102.83 ± 1.98	110.42 ± 1.56	117.32 ± 1.75	128.83 ± 1.62**
	Rosiglitazone	101.53 ± 1.68	104.20 ± 0.93	104.95 ± 1.17	110.03 ± 0.76##
	GGDGT	111.25 ± 1.32	106.80 ± 1.02	106.90 ± 0.95	112.15 ± 0.82##

Body weight expressed the mean of absolute weight of mice. ApoE-/-, apolipoprotein E knockout mouse; Rosiglitazone, WD + 10 mg/day/kg rosiglitazone; GGDGT, WD + 200 mg/day/kg GGDGT. Values are expressed as mean ± S.E. values (n = 12). **p < 0.01, vs. RD-fed control mice; #p < 0.05, ##p < 0.01 vs. WD-fed ApoE-/- mice.

Treatment with GGDGT for 12 weeks prevented a high cholesterol-induced elevation in SBP. In addition, WD-fed ApoE-/- mice had significantly increased plasma blood glucose and insulin levels (Table 2) compared to RD-fed control mice. However, treatment with GGDGT and rosiglitazone clearly lessened the increase in blood glucose (p < 0.01) and insulin (p < 0.05) levels in WD-fed ApoE-/- mice. WD-fed ApoE-/- mice showed significantly increased body weight compared with RD-fed control mice, while weight gain was significantly less in mice treated with GGDGT. It should be noted that during the 12 weeks of the WD regimen, cumulative food and water intake among the four groups was not significantly different (p > 0.05) (Table 1).

**Table 2:** **Effects of GGDGT on lipid profile, leptin, and insulin in ApoE-/- mice**

	Control	ApoE -/-	Rosiglitazone	GGDGT
Total cholesterol (mg/dl)	81.67 ± 4.98	393.00 ± 1.53***	118.00 ± 9.02###	349.67 ± 7.54##
Triglyceride (mg/dl)	53.67 ± 4.41	98.67 ± 2.40**	49.67 ± 2.33##	37.67 ± 2.85##
Leptin (mg/dl)	636.17 ± 20.5	2636.38 ± 41***	1362.5 ± 99.8###	986.33 ± 78.95###
Insulin (mg/dl)	2.28 ± 0.34	6.03 ± 0.63**	2.66 ± 0.48##	3.24 ± 0.79#

Values are expressed as mean **±** S.E. values (n = 12 mice per group). **p < 0.01, ***p < 0.001 vs. RD-fed control mice; ##p < 0.01, ###p < 0.001 vs. WD-fed ApoE-/- mice.

### GGDGT and lipid metabolism

Hypercholesterolemia is a common risk factor for early atherosclerosis. Prior to the appearance of atherosclerotic changes in the vascular wall, a high cholesterol level induces vascular functional changes that may lead to vascular remodelling and increases permeability of LDL-cholesterol [29]. Elevated total and LDL-cholesterol levels impair endothelial function, and LDL-cholesterol is deposited in the blood vessel wall as part of atherosclerotic plaques [30, 31]. However, increased HDL-cholesterol could suppress the atherosclerotic process by facilitating translocation of cholesterol from peripheral tissues like arterial walls to the liver for catabolism [24, 32]. In ApoE-/- mice, which lack the means to metabolize lipids, a fat-enriched diet can elevate the LDL-cholesterol level in plasma [33, 34]. Furthermore, ApoE-/- mice developed atherosclerosis, hypertension, impaired fasting glucose, impaired glucose tolerance, and modest dyslipidaemia. As expected, plasma leptin concentrations were also increased in mice that developed atherosclerosis [35]. In the present study, plasma levels of total cholesterol were significantly increased (p < 0.001) in WD-fed ApoE-/- mice; however, in mice treated with GGDGT and rosiglitazone, they were clearly decreased (p < 0.01). Triglyceride concentrations were increased in WD-fed ApoE-/- mice, whereas they were decreased (p < 0.01) in mice receiving rosiglitazone and GGDGT. In addition, leptin concentrations were markedly increased in WD-fed ApoE-/- mice compared to RD-fed control mice, while significantly decreased (p < 0.001) in mice receiving GGDGT (Table 2). These findings, at least in part, indicate that GGDGT may protect against the initiation and development of atherosclerosis by improving lipid metabolism.

### GGDGT and vascular morphology

**Figure 1:**
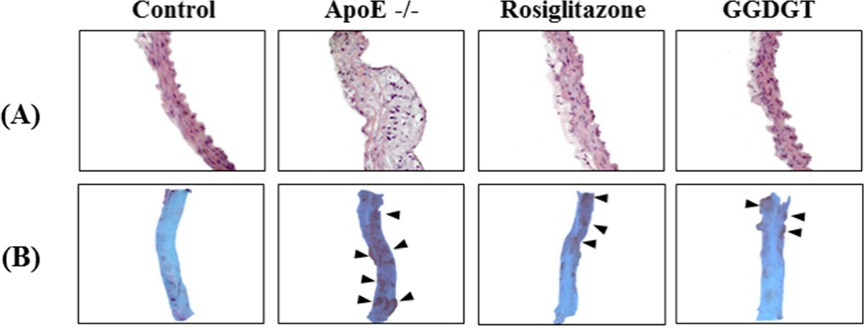
**Representative images showing aortic histology in control, ApoE-/-, rosiglitazone-treated, and GGDGT-treated mice at week 12.** The upper panel **(A)** indicates haematoxylin and eosin (H&E) staining (×400) in cross-section, and the lower panel **(B)** indicates Oil-red O staining (×100) (n = 6). The lipid accumulation (violet colour) is indicated by an arrow in the Oil-red O staining.

Endothelial dysfunction includes not only reduced vasodilation but also inflammation and atherosclerotic lesions [36, 37]. Blocking inflammatory mediators could decrease the size of atherosclerotic lesions. We hypothesized that the vasorelaxant effect of GGDGT would produce anti-inflammatory and anti-atherosclerotic effects in WD-fed ApoE-/- mice. Microscopic examination of H&E stained thoracic aortic sections revealed roughened endothelial layers in WD-fed ApoE-/- mice. The aortic sections of WD-fed ApoE-/- mice also showed significantly increased tunica intima thickness. Treatment with GGDGT and rosiglitazone for 12 weeks maintained the smooth and soft character of the intima endothelial layers and decreased intima-media thickness in aortic sections. Moreover, WD-fed ApoE-/- mice were observed to have larger atherosclerotic lesions, whereas atherosclerotic lesions were not observed in mice treated with GGDGT and rosiglitazone (Figure 1A). In addition, oil-red O staining of atherosclerotic plaques is visible as a red colour. Figure 1B shows the proximal portion of the descending thoracic aorta that has been turned inside-out to expose the stained endothelium. The surface area covered with atherosclerotic plaque was statistically higher in the aortas of WD-fed ApoE-/- mice than in RD-fed control mice, whereas aortas from mice treated with GGDGT and rosiglitazone did not show significant atherosclerotic lesions. Previous histological analysis has demonstrated that rougher intimal endothelial layers in aortic sections of WD-fed ApoE-/- mice were associated with a trend towards a thickened medial layer [38, 39]. Thus, feeding ApoE-/- mice a fat-enriched WD could induce thickening of the aortic intima-media that is compatible with the processes of atherosclerosis and intimal derangement, and our experiments showed that these morphological changes could be prevented by GGDGT treatment.

### Effects of GGDGT on endothelial dysfunction: vascular relaxation

**Figure 2:**
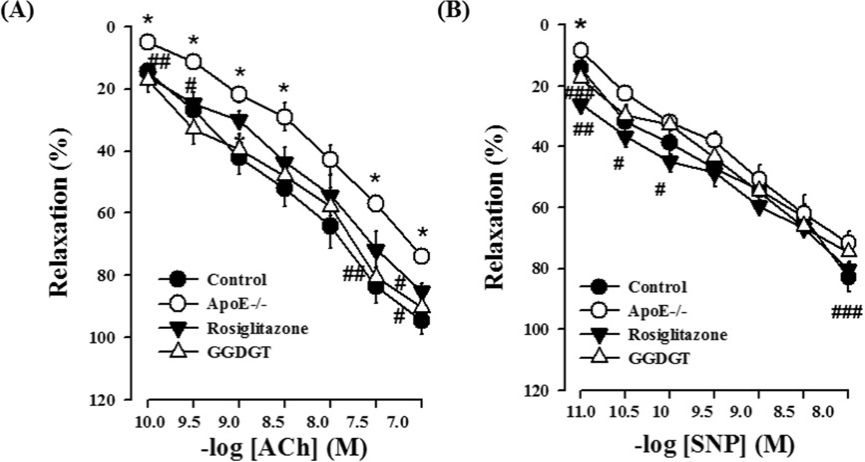
**Effect of GGDGT on relaxation of the thoracic aorta in Western diet (WD)-fed ApoE-/- mice. (A)** Aortic relaxation induced by acetylcholine (Ach), and **(B)** aortic relaxation induced by sodium nitroprusside (SNP). Data are mean ± S.E. values (n = 10 mice per group). *p < 0.05, **p < 0.01 vs. regular diet-fed control mice; #p < 0.05, ##p < 0.01, ###p < 0.001 vs. WD-fed ApoE-/- mice.

**Figure 3:**
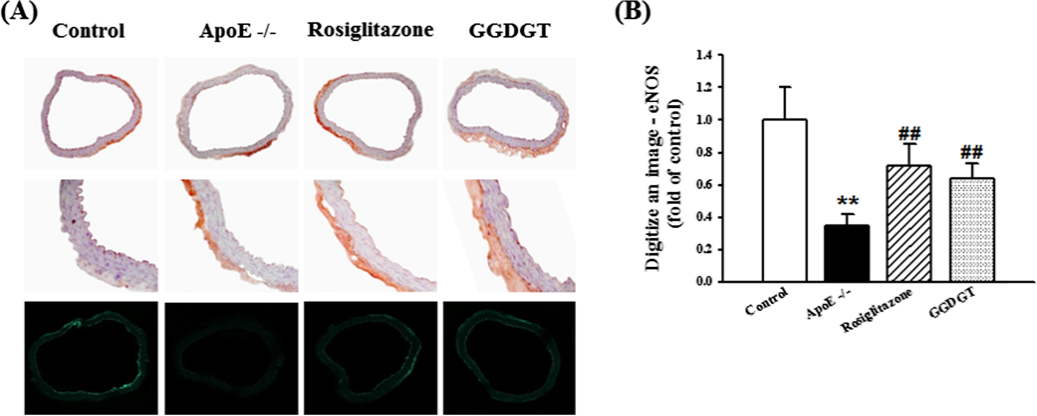
**Effect of GGDGT on eNOS expression in the aorta of Western diet (WD)-fed ApoE-/- mice. (A)** Representative microscopic photographs of aortas immunodetected for endothelial nitric oxide synthase (eNOS) (upper panels, magnification × 100; middle panels, magnification × 400; lower panels, magnification × 100). **(B)** Quantification of eNOS expression. Aortas were obtained from regular diet (RD)-fed control mice, WD-fed ApoE-/- mice, rosiglitazone-treated mice, and Gal-geun-dang-gwi-tang (GGDGT)-treated mice. Values are expressed as a percentage of the density of the blot coloured dark brown (mean ± S.E.) (n = 6 mice per group). **p < 0.01 vs. RD-fed control mice; ##p < 0.01 vs. WD-fed ApoE-/- mice.

The endothelium can sense changes or abnormalities in blood flow and pressure, and the vascular endothelium exists between circulating blood and vascular smooth muscle cells, while the vascular smooth muscle plays the important role of modulating vascular tone [40]. Recent studies showing improvements in insulin-regulated vascular homeostasis through the restoration of vascular constriction in various animal models suggest a relation between insulin signalling and vascular dysfunction [41–43]. Figure 2 shows the effect of GGDGT on vasodilatory response to ACh or SNP in aortic strips with endothelium from the WD-fed ApoE-/- mice. The relative relaxation induced by ACh was attenuated in thoracic aortas taken from the mice fed an atherogenic diet compared with that of the control group (p < 0.05). ACh-induced vascular relaxation in the aortas taken from GGDGT treated WD-fed ApoE-/- mice was in some respects similar to that of RD-fed control mice (Figure 2A). However, vasodilation in response to low concentrations of SNP, an exogenous NO donor, was not significantly decreased by treatment with GGDGT and rosiglitazone for 12 weeks (Figure 2B). Furthermore, Figure 3 displays representative pictures of eNOS expression in thoracic aortic sections of WD-fed ApoE-/- mice using immunohistochemistry. The expression of eNOS was suppressed in the aortas of WD-fed ApoE-/- mice compared with RD-fed control mice. Treatment with GGDGT and rosiglitazone markedly restored expression levels of eNOS by 44% and 65%, respectively. These finding suggest that the hypotensive effect of GGDGT is mediated by ACh and via the endothelium-dependent NO-cGMP pathway. In fact, endothelial dysfunction was initially identified as impaired vasodilation to specific stimuli such as ACh or bradykinin, therefore, improvement of endothelial function is predicted to regulate lipid homeostasis [44]. Impairment of ACh-induced relaxation of the aorta was observed in obese, diabetic, fatty rats because of endothelial dysfunction [45]. It has been well documented that endothelium-dependent vascular relaxation is abnormal in both hypercholesterolemia and atherosclerosis because of NO’s role in maintaining vascular tone [46, 47]. Thus, our finding suggests that GGDGT plays a protective role in diet-induced hypertension and vasoconstriction. Further studies are required to measure plasma nitrate/nitrite levels and vascular relaxation by SNP after Ach, which is the alleviation of endothelial damage in vascular dysfunction seen with GGDGT treatment.

### GGDGT and vascular inflammatory markers: ICAM-1 expression

**Figure 4:**
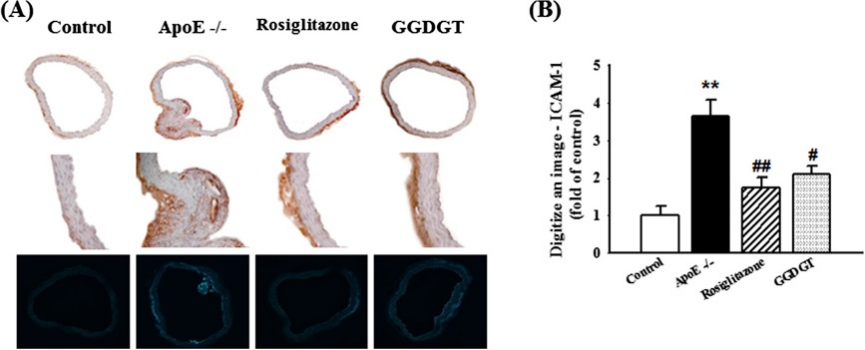
**Effect of GGDGT on ICAM-1 immunoreactivity in the aorta of Western diet (WD)-fed ApoE-/- mice. (A)** Immunohistochemical staining of ICAM-1 in aortas from regular diet (RD)-fed control mice, WD-fed ApoE-/- mice, ApoE-/- mice treated with rosiglitazone, and ApoE-/- mice treated with Gal-geun-dang-gwi-tang (GGDGT) (upper panels, magnification × 100; middle panels, magnification × 400; lower panels, magnification × 100). **(B)** Quantitative analysis of ICAM-1 immunoreactivity in the thoracic aorta. The average score for 5–10 randomly selected aortas was calculated. Data are expressed as mean ± S.E. values (n = 6 mice per group). **p < 0.01 vs. RD-fed control mice; #p < 0.05, ##p < 0.01 vs. WD-fed ApoE-/- mice.

**Figure 5:**
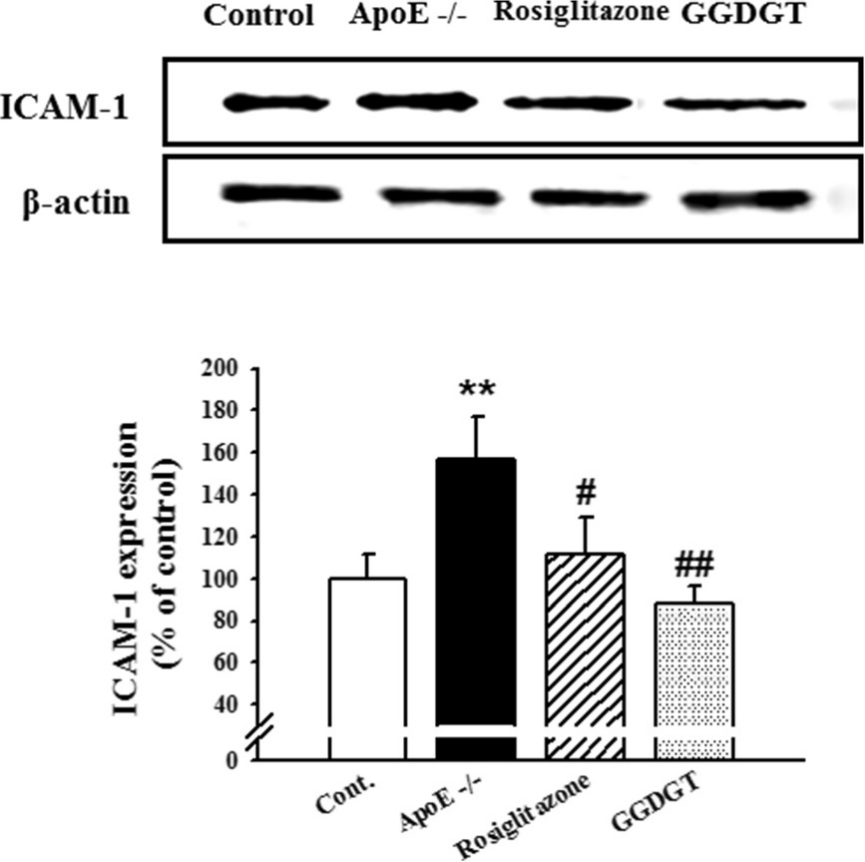
**Effect of GGDGT on expression of ICAM-1 in the aortas of WD-fed ApoE-/- mice.** Western blots and corresponding densitometric analysis of intercellular adhesion molecule (ICAM)-1 expression in aortic tissue. Data are expressed as a percentage of the density of blots and are mean ± S.E. values (n = 3 mice per group). **p < 0.01 vs. regular diet (RD)-fed control mice; #p < 0.05, ##p < 0.01 vs. western diet (WD)-fed ApoE-/- mice.

Reduced vasodilation, inflammation, and atherosclerotic lesions are associated with endothelial dysfunction [36, 37]. Activation of the endothelium at sites of inflammation allows numerous leukocytes to adhere to the vascular endothelium, transmigrates the endothelium, and aggravates endothelial dysfunction and tissue injury [48]. In leukocyte infiltration, the sites of inflammation are regulated in part by specific endothelial-leukocyte adhesion molecules, including vascular cell adhesion molecule-1 (VCAM-1), ICAM-1, and E-selectin [49]. In a previous study, we investigated the effect of Gal-geun-dang-gwi-tang in suppressing the expression of cell adhesion molecules, such as VCAM-1, ICAM-1, and E-selectin, in vascular endothelial cells stimulated by high glucose. Pre-treatment with Gal-geun-dang-gwi-tang significantly inhibited high glucose-induced expression of VCAM-1, ICAM-1, and E-selectin in a dose-dependent manner (Additional file 1: Figure S1). Therefore, we chose this herbal prescription to investigate diabetic vascular complications in a murine model. We hypothesized that the vasorelaxant effect of GGDGT would contribute anti-inflammatory and anti-atherosclerotic effects in mice fed a fat-enriched WD. In this study, analysis of immunohistochemical staining showed that ICAM-1 was weakly expressed in the thoracic aortas of the RD-fed control mice, but was markedly increased in WD-fed ApoE-/- mice. In contrast, ICAM-1 expression was significantly decreased by treatment with GGDGT (p < 0.05) (Figure 4). Expression levels of endothelial ICAM-1 in the thoracic aorta were determined by western blotting analysis. The WD-fed ApoE-/- mice showed significantly increased aortic expression levels of ICAM-1 compared with RD-fed control mice, whereas treatment with GGDGT and rosiglitazone for 12 weeks showed a lower band intensity of ICAM-1 compared with WD-fed ApoE-/- mice (60% decrease) (Figure 5). Additionally, VCAM-1 and E-selectin immunoreactivity was increased in the aortas of WD-fed ApoE-/- mice. However, VCAM-1 and E-selectin expression was significantly decreased by treatment with GGDGT (p < 0.05 and 0.01, respectively) (Additional file 1: Figures S2 and S3). These finding suggest that GGDGT may have a potentially important role in anti-inflammatory and anti-atherosclerotic activity in diabetic vascular dysfunction.

### Effects of GGDGT on glucose tolerance and insulin receptor expression in skeletal muscle

**Figure 6:**
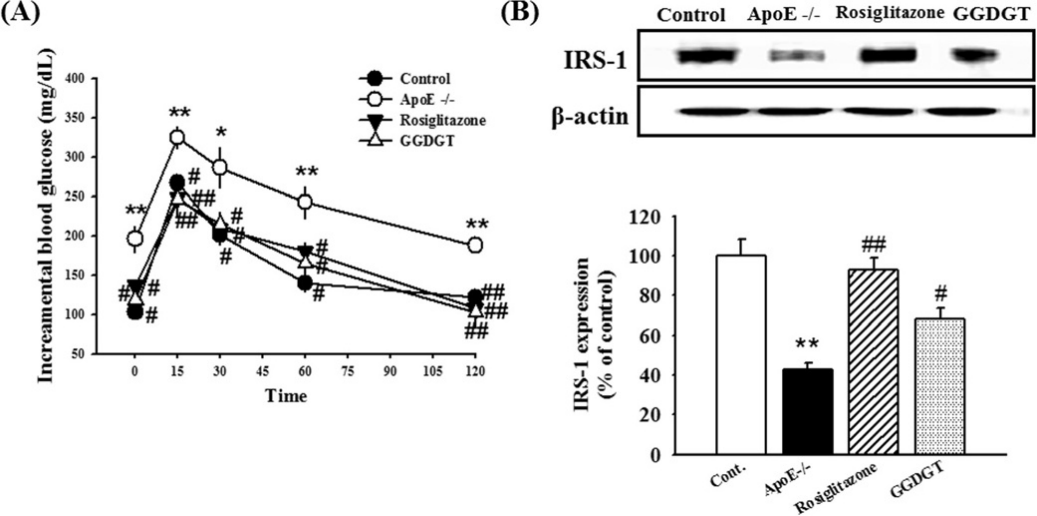
**Effect of GGDGT on the OGTT and expression of insulin receptors in the muscle tissue. (A)** Mice were fasted overnight and glucose (1.5 g/kg) was administered for an oral glucose tolerance test (OGTT). **(B)** Western blots and corresponding densitometric analysis of insulin receptor substrate (IRS)-1 expression in the muscle tissue. Data are expressed as mean ± S.E. values (n = 3 mice per group). *p < 0.05, **p < 0.01 vs. regular diet (RD)-fed control mice; #p < 0.05, ##p < 0.01 vs. Western diet (WD)-fed ApoE-/- mice.

Insulin resistance plays an important role in the development of abnormalities such as impaired glucose tolerance, type 2 diabetes, obesity, and hyperlipidemia [50]. In the present study, an oral glucose tolerance test (OGTT) was performed on WD-fed ApoE-/- mice. As shown in Figure 6A, the mice were fasted overnight before glucose was administered (1.5 g/kg). Over the entire time course, blood glucose levels were higher in RD-fed control mice than in WD-fed ApoE-/- mice; however, treatment with GGDGT and rosiglitazone for 12 weeks further decrease blood glucose levels in ApoE-/- mice compared with RD-fed control mice (p < 0.01 and p < 0.01, respectively). Both the rise and fall of blood glucose levels were slower in WD-fed ApoE-/- mice than in mice that were treated with GGDGT. Insulin signalling from the insulin receptor is transmitted through insulin receptor substrate (IRS)-1. IRS-1 tyrosine phosphorylation has been implicated in signal transduction from the insulin receptor to phosphatidylinositol 3-kinase, leading to GLUT-4 translocation and subsequent glucose uptake [51–53]. To determine when and to what extent skeletal muscle tissues become insulin insensitive, we investigated insulin signalling, a key molecular event in the pathogenesis of fat-induced insulin resistance in vivo. As shown in Figure 6B, WD-fed ApoE-/- mice showed significantly decreased IRS-1 expression compared with RD-fed control mice, whereas mice receiving GGDGT and rosiglitazone recovered normal expression of IRS-1. GGDGT improved glucose tolerance, restored the expression of IRS-1 in skeletal muscle, and decreased plasma insulin levels. These results suggest that GGDGT ameliorates insulin resistance and that its action is related to the insulin-signalling pathway [54]. Furthermore, the results suggest that GGDGT ameliorated systemic vascular dysfunction, including vasoconstriction and inflammation, through activation of insulin signalling in WD-fed ApoE-/- mice.

## Conclusions

Gal-geun-dang-gwi-tang has long been used for treatment of vascular disorders, but the pharmacologic mechanisms of the herbal combination are unknown. Treatment of WD-fed ApoE-/- mice with GGDGT reduced hypertension by protecting the endothelium-dependent vasorelaxation response. GGDGT also improved total cholesterol and triglyceride levels and reduced expression of the vascular inflammation marker, ICAM-1. Furthermore, GGDGT ameliorated insulin resistance by decreasing plasma levels of insulin, improving glucose tolerance, and restoring insulin signalling by recovery of IRS-1 expression in skeletal muscle tissues. Accordingly, the Korean medicine prescription GGDGT may be useful in the treatment and prevention of diabetic vascular complications. To our knowledge, this study is the first to demonstrate the apparent anti-diabetic, anti-hypertensive, hypolipidemic, and vascular anti-inflammatory effects of GGDGT in an animal model of diabetic atherosclerosis.

## Electronic supplementary material

Additional file 1: Figure S1: Effect of GGDGT on high glucose (HG)-induced ICAM-1, VCAM-1, and E-selectin expression in the HUVEC. **Figure S2.** Effect of GGDGT on VCAM-1 expression in the aorta of WD-fed ApoE-/- mice. **Figure S3.** Effect of GGDGT on E-selectin expression in the aorta of WD-fed ApoE-/- mice. (DOC 792 KB)

## Abbreviations

GGDGT: Gal-geun-dang-gwi-tang
ApoE-/-: Apolipoprotein E knockout
LDL: Low-density lipoprotein
HDL: High-density lipoprotein
RD: Regular diet
WD: Western diet
SBP: Systolic blood pressure
NO: Nitric oxide
ENOS: Endothelial NO synthase
ACh: Acetylcholine
SNP: Sodium nitroprusside
ICAM-1: Intercellular adhesion molecule 1
VCAM-1: Vascular cell adhesion molecule-1
IRS-1: Insulin receptor substrate-1
OGTT: Oral glucose tolerance test.
