# Supplementary material for: Gal-geun-dang-gwi-tang improves diabetic vascular complication in apolipoprotein E KO mice fed a western diet
Source: BMC Complement Altern Med. 2014 Nov 22;14:453. doi: 10.1186/1472-6882-14-453 (PMC4247676; doi:10.1186/1472-6882-14-453)
Supplement: Supplementary file 1 — Additional file 1: Figure S1: Effect of GGDGT on high glucose (HG)-induced ICAM-1, VCAM-1, and E-selectin expression in the HUVEC. Figure S2. Effect of GGDGT on VCAM-1 expression in the aorta of WD-fed ApoE-/- mice. Figure S3. Effect of GGDGT on E-selectin expression in the aorta of WD-fed ApoE-/- mice. (DOC 792 KB) [file 12906_2014_2024_MOESM1_ESM.doc]

**Gal-geun-dang-gwi-tang improves insulin resistance and vascular dysfunction in apolipoprotein E KO mice fed a western diet**

1. **Materials and Methods**

**Cell cultures**

Primary cultured HUVEC and endothelial cell growth medium (EGM-2) containing 2.5% fetal bovine serum (FBS) and growth supplements were purchased from Cambrex (East Rutherford, NJ). HUVEC which were used between passages 3 and 8 were maintained in EGM-2 in a humidified chamber containing 5% CO_2_ at 37°C.

**Determination of cell based ELISA**

ELISA was used to determine the level of ICAM-1, VCAM-1, and E-selectin expression on the cell surface, as previously described with minor modifications. Briefly, HUVEC were fixed by 1% paraformaldehyde and exposed to mouse anti-human ICAM-1, VCAM-1, or E-selectin antibodies at 1:1000 dilution in the phosphate-buffered saline (PBS) containing 1% bovine serum albumin (BSA) for 2 h at room temperature. The cells were washed and incubated with a horseradish peroxidase (HRP)-conjugated secondary antibody. The expression of VCAM-1, ICAM-1, or E-selectin was quantified by adding a peroxidase substrate solution (40 mg o-phenylenediamine and 10 μL 30% H_2_O_2_ in 100 mL 0.05 M citrate-phosphate buffer). After incubation for 30 min at 37°C, the reaction was stopped by addition of 5 N H_2_SO_4_, and the absorbance of each well was measured at 490 nm by a Multiskan microplate reader (Thermo LabSystems Inc., Franklin, MA).

1. **Results**

Firstly, we performed ELISA and western blotting analysis to investigate the effect of Gal-geun-dang-gwi-tang in suppressing cell adhesion molecules such as vascular cell adhesion molecule-1 (VCAM-1) and intracellular adhesion molecule-1 (ICAM-1), and E-selectin expression in high glucose stimulated human umbilical vein endothelial cells (HUVEC). Pretreatment with Gal-geun-dang-gwi-tang was significantly inhibited the high glucose (HG)-induced VCAM-1, ICAM-1, and E-selectin expression in a dose dependent manner (Supple Fig. 1). Therefore, we choice this herbal medicine, that it will be selected, performing animals experiments.

In supple Fig. 2 and 3, VCAM-1 and E-selectin immunoreactivity was increased in aorta of WD-fed ApoE-/- mice. However, VCAM-1 and E-selectin expression was significantly decreased by treatment with GGDGT (p < 0.05, 0.01, respectively).


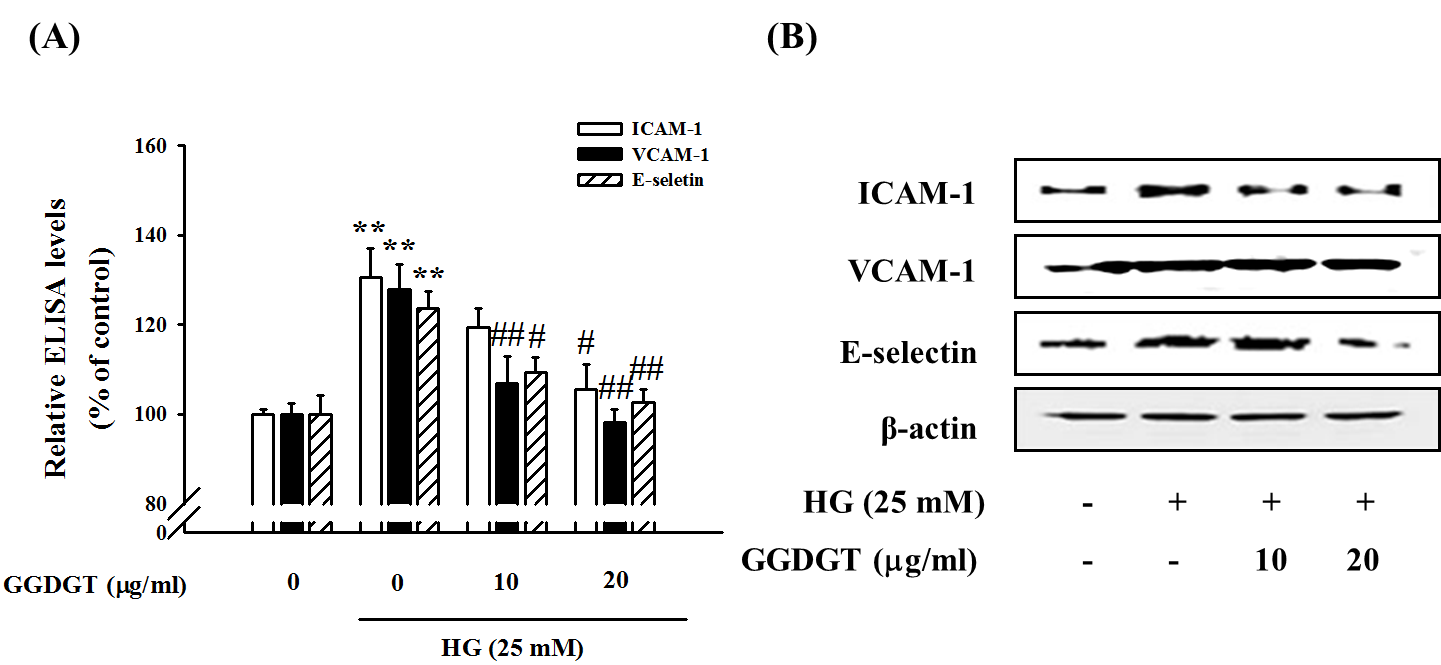


**Figure S1.** **Effect of GGDGT on high glucose (HG)-induced ICAM-1, VCAM-1, and E-selectin expression in the HUVEC.** (A) HUVEC surface expressions of intercellular adhesion molecule-1 (ICAM-1), vascular cell adhesion molecule-1 (VCAM-1), and E-selectin were analyzed by cell-based ELISA. The results are expressed as a percentage of HG-induced expression (mean ± S.E.) of three individual experiments. **p < 0.01 vs. control, #p < 0.05, ##p < 0.01 vs. HG. (B) Cells were pretreated with GGDGT for 30 min and then stimulated with HG (25 mM) for 24 h. The whole protein was extracted in HUVEC by Western blot analysis.


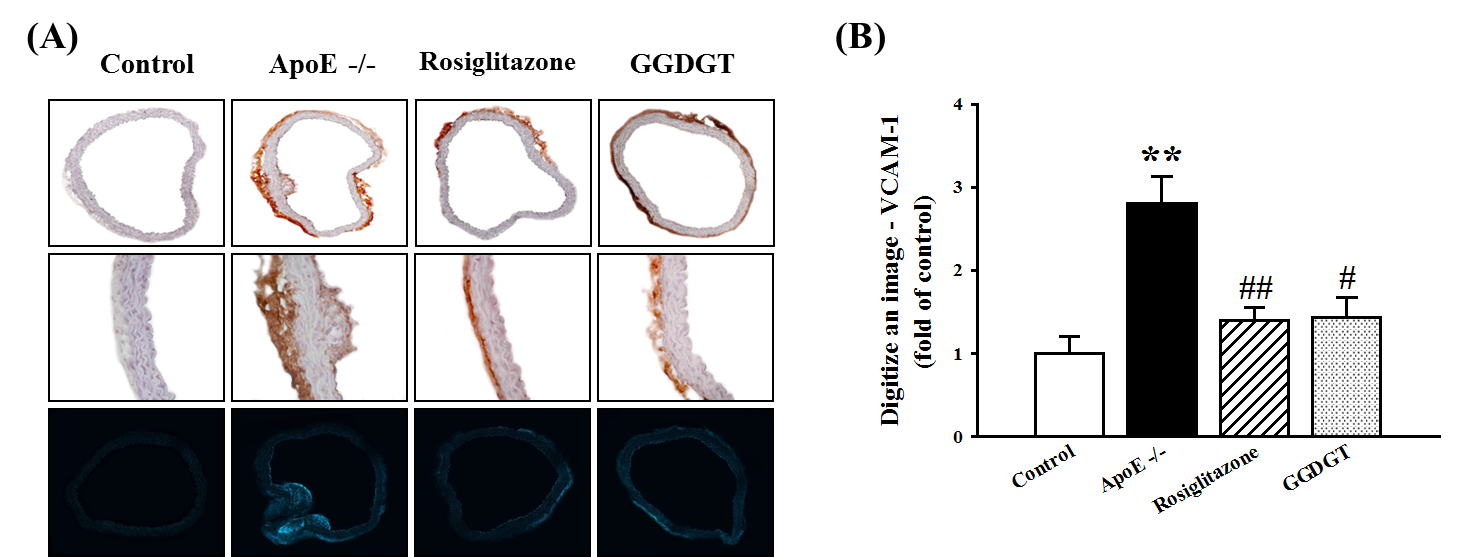


**Figure S2. Effect of GGDGT on VCAM-1 expression in the aorta of WD-fed ApoE-/- mice.** (A) Representative microscopic photographs of the aorta immunodetected with VCAM-1, and (B) quantifications were shown. Aortae were obtained from RD-fed control mice, WD-fed ApoE-/- mice, rosiglitazone, and GGDGT. Values are expressed as a percentage of the density of blot colored dark brown (mean ± S.E.) (n=6 mice per group). **p < 0.01 vs. RD-fed control mice; #p < 0.05, ##p < 0.01 vs. WD-fed ApoE-/- mice.


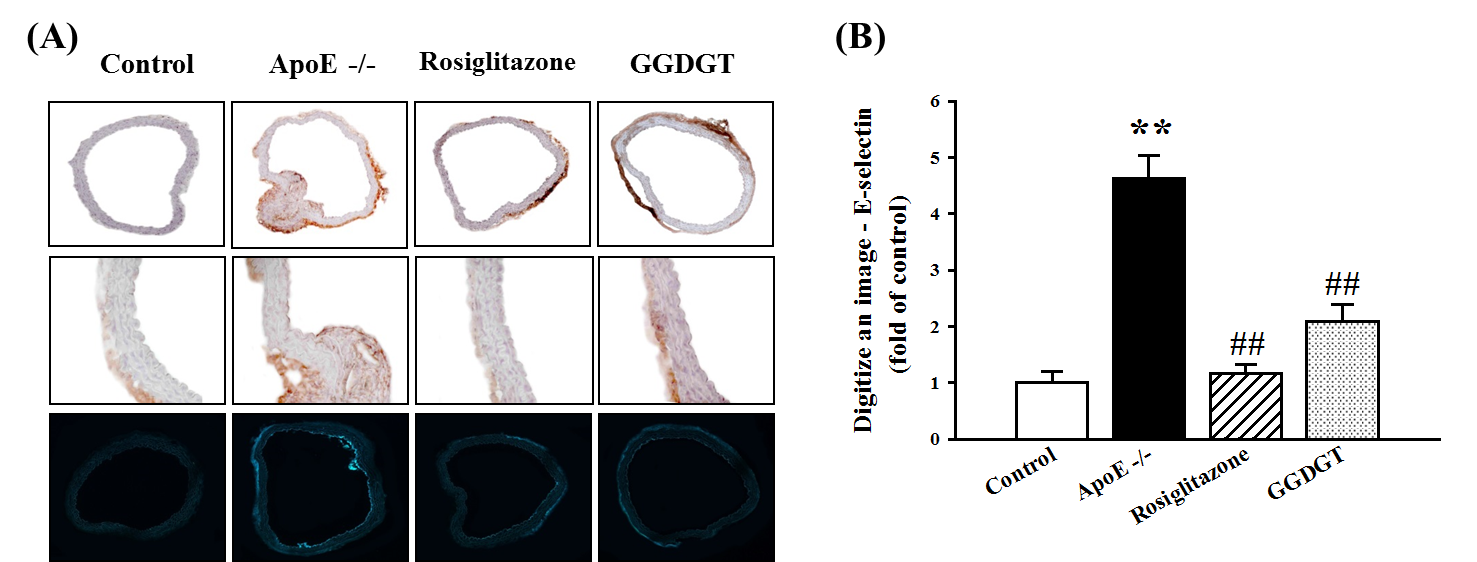


**Figure S3.** **Effect of GGDGT on E-selectin expression in the aorta of WD-fed ApoE-/- mice.** (A) Representative microscopic photographs of the aorta immunodetected with E-selectin, and (B) quantifications were shown. Aortae were obtained from RD-fed control mice, WD-fed ApoE-/- mice, rosiglitazone, and GGDGT. Values are expressed as a percentage of the density of blot colored dark brown (mean ± S.E.) (n=6 mice per group). **p < 0.01 vs. RD-fed control mice; ##p < 0.01 vs. WD-fed ApoE-/- mice.
